# Supplementary material for: Yu–Shiba–Rusinov screening of spins in double quantum dots
Source: Nat Commun. 2018 Jun 18;9:2376. doi: 10.1038/s41467-018-04683-x (PMC6006160; doi:10.1038/s41467-018-04683-x)
Supplement: Supplementary file 1 — Supplementary Information [file 41467_2018_4683_MOESM1_ESM.pdf]

Supplementary Information
Yu-Shiba-Rusinov screening of spins in double quantum dots

CONTENTS

Supplementary Note 1: Theoretical model 2

Supplementary Note 2: Additional data on N-DQD-S systems 4

Device A 4

Device B 7

Supplementary Note 3: Zero-bandwidth approximation 10

Spectral weight of the triplet state 14

Supplementary Note 4: Sub-gap conductance via YSR states 15

Bias asymmetry and quasiparticle relaxation 17

Bias asymmetry at the particle-hole symmetric point. 20

Supplementary References 22

SUPPLEMENTARY NOTE 1: THEORETICAL MODEL

The double-dot system is described by a two-orbital Anderson model,

$$H = H_{\mathrm{L}} + H_{\mathrm{D}} + H_{\mathrm{T}} \tag{1}$$

where L refers to the leads, D to the dots, and T to the tunnel coupling between the different parts. Since our system has the two dots in series, we shall refer to them by either $\mathrm{QD_N}$ or $\mathrm{QD_S}$, or sometimes even just N or S, i.e. with the label of the closest N (normal) or S (superconducting) lead (cf. Supplementary Fig. 1). The three parts of the Hamiltonian are

$$H_{\mathrm{D}} = \sum_{i=\mathrm{S/N}} \left[\sum_{\sigma} \epsilon_i n_{i\sigma} + U_i n_{i\uparrow} n_{i\downarrow} \right] + \sum_{\sigma\sigma'} U_{\mathrm{d}} n_{\mathrm{N}\sigma} n_{\mathrm{S}\sigma'}, \tag{2}$$

$$H_{\mathrm{L}} = \sum_{\alpha=\mathrm{N/S};k} \left[\sum_{\sigma} \xi_{\alpha k} c^{\dagger}_{\alpha k\sigma} c_{\alpha k\sigma} + \Delta_{\alpha} (c^{\dagger}_{\alpha k\uparrow} c^{\dagger}_{\alpha -k\downarrow} + c_{\alpha -k\downarrow} c_{\alpha k\uparrow}) \right], \tag{3}$$

$$H_{\mathrm{T}} = t_{\mathrm{S}} \sum_{k\sigma} c^{\dagger}_{\mathrm{S}k\sigma} d_{\mathrm{S}\sigma} + t_{\mathrm{d}} \sum_{\sigma} d^{\dagger}_{\mathrm{S}\sigma} d_{\mathrm{N}\sigma} + t_{\mathrm{N}} \sum_{k\sigma} c^{\dagger}_{\mathrm{N}k\sigma} d_{\mathrm{N}\sigma} + h.c., \tag{4}$$

where $\Delta_{\mathrm{N}} = 0$ and $\Delta_{\mathrm{S}} = \Delta$ denotes the (BCS) superconducting gap in lead S, and $\xi_{\alpha k}$ denotes the energy relative to the chemical potential of an electron with momentum k in lead α. Operators $c^{(\dagger)}_{\alpha k\sigma}$ and $d^{(\dagger)}_{i\sigma}$ annihilate(create) electrons in respectively leads and dots, and operators $n_{i\sigma} = d^{\dagger}_{i\sigma} d_{i\sigma}$ count the number of electrons with spin σ on dot i. Individual dot energies, ϵ_i, are controlled by individual gate voltages, and intra, and inter-dot Coulomb interaction are denoted by $U_{\mathrm{S/N}}$, and U_{d} respectively. Finally, dot-lead, and inter-dot tunneling amplitudes are denoted respectively by $t_{\mathrm{S,N}}$ and t_{d}.

Since our systems have $U_{\mathrm{S}}, U_{\mathrm{N}} \gtrsim \Delta$, Coulomb interactions impede proximity effect in the dots and instead one should observe Yu-Shiba-Rusinov (YSR) states for charge states with a

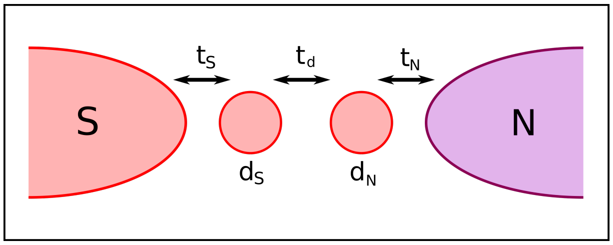

Supplementary Figure 1. Device model. Schematic of the double dot system with the superconducting lead on the left and the normal lead on the right. Parameters t_{d}, t_{S} and t_{N} denote the relevant tunnel couplings.

finite dot spin [1]. Since also $t_{\mathrm{S}} \gg t_{\mathrm{N}}$, we expect the Kondo temperature due to the normal lead to be very small, and throughout our analysis, we shall merely treat the normal lead as a weakly coupled tunnel probe.

SUPPLEMENTARY NOTE 2: ADDITIONAL DATA ON N-DQD-S SYSTEMS

This section presents additional data on normal metal-double quantum dot-superconductor (N-DQD-S) systems for two devices (A and B). We first elaborate on the data set in Fig. 3 in the partly screened regime, which was measured in device A. In particular, we clarify how the parameters used in the fit of the zero-band-width (ZBW) model are estimated by analyzing stability diagrams and bias spectroscopy plots. Furthermore, we comment on the behavior of other double-dot shells observed in the stability diagram. Finally, we present stability diagrams and sub-gap spectroscopy plots for device B in the honeycomb regime, where the tunnel couplings between the dots and to the superconductor are small.

Device A

Supplementary Fig. 2 shows the linear conductance of a gate region where two (three) orbitals are filled as the voltage on gate g_S (g_N) is swept in the superconducting state. Depending on the DQD-S parameters (which are tuned by the gate) different types of behavior

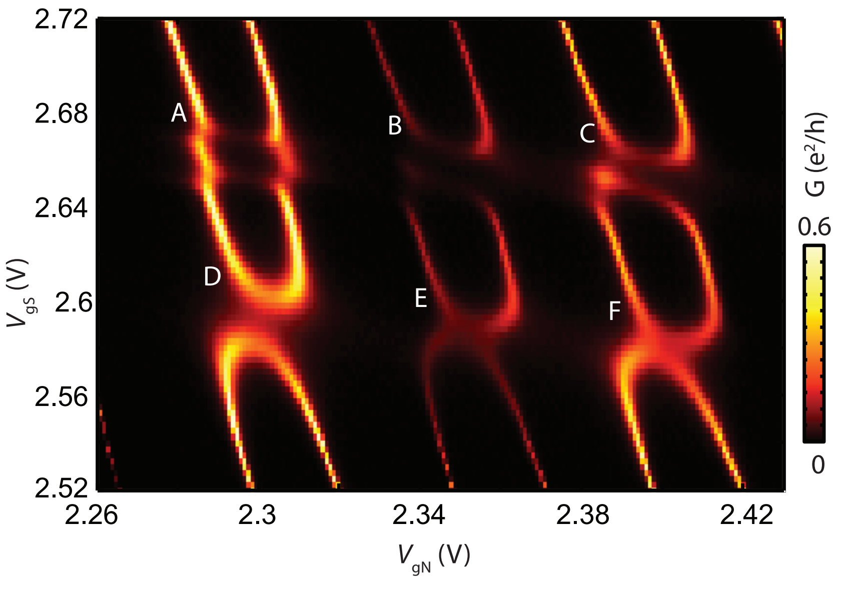

Supplementary Figure 2. Device A (shells A-F). Stability diagram at 30 mK showing the filling of 2×3 orbitals (double-dot shells) in the superconducting state. For these gate voltages, two-orbital double dot shells in the honeycomb (A) and partly screened (in particular D-F) regimes are observed due to a change in parameters as electrons are added to the orbitals of the two dots.

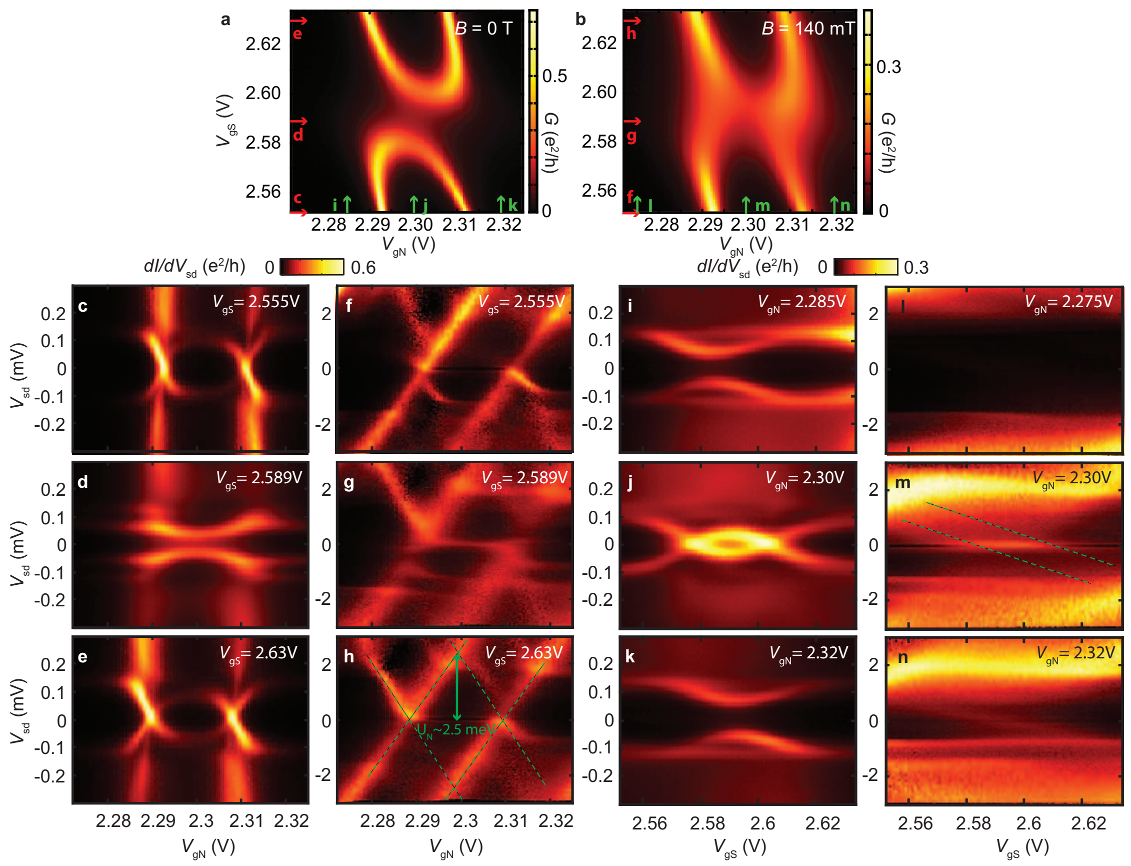

Supplementary Figure 3. Device A (shell D). Additional plots for parameter estimation. **a**, Linear conductance versus plunger gates in the superconducting state. **b**, Stability diagram in the normal state ($B = 140\,\mathrm{mT}$). The smeared features are attributed to the strong coupling to S. **c-e**, Sub-gap spectroscopy plots of $\mathrm{QD_N}$ in the partly screened regime (also shown in Fig. 3c-e). **f-h**, Bias spectroscopy plots in the normal state ($B = 120\,\mathrm{mT}$) corresponding to (**c-e**) showing Coulomb blockade diamonds in $\mathrm{QD_N}$ yielding $U_\mathrm{N} \sim 2.5\,\mathrm{meV}$ (see green dashed lines in **h**). **i-k**, Sub-gap spectroscopy plots of $\mathrm{QD_S}$ identical to Fig. 3. **l-n**, High bias stability diagrams ($B = 120\,\mathrm{mT}$) corresponding to **i-k** showing faint (Coulomb blockade) features. A Coulomb energy in the order of $U_\mathrm{S} \sim 0.8\,\mathrm{meV}$ is estimated. The estimate is found via the dashed lines in **m** aligned to the Kondo-like feature with slopes estimates from the slanting feature in **n**. Note, that **i** and **l** are measured at a slightly different V_gN.

are observed. The two-orbital double-dot shell A in the top left corner shows a honeycomb diagram representing the familiar honeycomb regime (see ZBW model calculations in Fig. 2f in the article). Interestingly, the stability diagram also shows other two-orbital shells,

Device	Regime	U_{N} (meV)	U_{S} (meV)	U_{d} (meV)	t_{d} (meV)	t_{S} (meV)	Δ (meV)
A (shell D)	PS	2.5	0.8	0.1	0.27	0.22	0.14
B	HC	3.1	2.9	0.8	0.01	0.1	0.14

Supplementary Table 1. Parameters used in model. Parameters used for the zero-bandwidth model for device A in the partly screened regime and device B in the deep honeycomb regime.

in particular D-F, which all resemble the behaviour of the partly screened regime. The double-dot shell D was analyzed in Fig. 3 in the article. The double-dot shells B-C display less regular behaviour, which we interpret as the behaviour expected close to the transition between the honeycomb and partly screened regimes (see for instance the ZBW and NRG generated stability diagrams versus tuning of t_{S} in Supplementary Figs. 7c,f and 7g,j).

First we look at shell D in the partly screened regime in more detail. In Supplementary Fig. 3a-b stability diagrams (linear conductance versus plunger gates) are shown in the superconducting and normal ($B = 140\,\mathrm{mT}$) states. In the normal state (Supplementary Fig. 3b), the features are smeared, which is attributed to the strong coupling to the superconducting electrode. We note that in studies of the two-impurity Kondo effect with double dots strongly coupled to leads, the honeycomb pattern appears similarly smeared [2]. When the electrode turns superconducting, the features sharpen and a conductance pattern resembling the partly screened regime is observed (Supplementary Fig. 3a). Similar characteristic mirrored arc patterns have also been observed in SQUID nanotube samples in the S-DQD parallel configuration (where these patterns are also expected for appropriate parameters) [3].

The parameters used in the ZBW modelling are shown in Supplementary Table 1. We now explain how they were obtained. For easy comparison, columns 1 and 3 (c-e & i-k) in Supplementary Fig. 3 show sub-gap spectroscopy plots, also displayed in the main text in Fig. 3, while column 2 and 4 (f-h & l-n) show the corresponding bias spectroscopy plots for high bias in the normal state ($B = 140\,\mathrm{mT}$). For $\mathrm{QD_N}$, the high bias spectroscopy plots (f-h) reveal clear Coulomb blockade diamond structure and a Coulomb energy in the order of $U_{\mathrm{N}} \sim 2.5\,\mathrm{meV}$ is estimated. In the case of $\mathrm{QD_S}$, the high bias spectroscopy plots (l-n) are more difficult to interpret due to the strong coupling to S. We estimate a Coulomb energy in the order of $0.8\,\mathrm{meV}$. The dashed lines in Supplementary Fig. 3m represent the slope of

the features in Supplementary Fig. 3n aligned to the Kondo-like features in the data, which yields an upper limit of around 1 meV. Since the important parameter for the model is t_S^2/U_S (not U_S), an error in the estimate of the charging energy may be compensated by adjusting the tunnel coupling t_S, and therefore we do not have a fully independent estimate for this parameter. We use t_S and t_d as fitting parameters to fit the 6 sub-gap spectroscopy plots in Fig. 3 for one set of parameters. Finally, due to the strong coupling of the superconducting leads, the normal state honeycomb diagram appears smeared, which makes it difficult to estimate U_d in contrast to the honeycomb regime below. In the fitting, we used $U_d \sim 0.1$ meV. The parameters used in the modelling are summarized in Supplementary Table 1.

Device B

Supplementary Fig. 4 shows a dataset for the device B in the honeycomb regime. Supplementary Fig. 4a and 4b display the conductance versus plunger-gate voltages in the superconducting and normal states, respectively. Interestingly, at finite bias in the superconducting state, identification of sub-gap resonances corresponding to QD_S (the dot attached to the superconducting electrode) can easily be made, since the sub-gap excitations lead to a splitting of the zero-bias feature at finite bias (see green arrows in Supplementary Fig. 4c). Gap spectroscopy along the dashed lines shown in Supplementary Fig. 4a are displayed in Supplementary Fig. 4d-i. All panels show qualitatively similar behaviour, confirming the appearance of sub-gap excitations only in QD_S. This observation is attributed to a low tunnel coupling t_d between the two dots and consistent with the intuition that superconductivity affects the closest dot most strongly. ZBW modelling of the sub-gap state behaviour through the (11) region is shown in Supplementary Fig. 4j-k giving a qualitatively good correspondence with the experimental data in Supplementary Fig. 4e,h. In this case, the estimation of parameters are more straightforward than in the partly screened regime due to the sharp features in the normal state data. In particular, we have used the normal state stability diagram of Supplementary Fig. 4b and bias spectroscopy plots at large bias to obtain the parameters [4].

Supplementary Fig. 5 shows high bias spectroscopy plots along the red and green lines in Supplementary Fig. 4a, i.e. altering the occupation of QD_N and QD_S, respectively. Clear Coulomb blockade diamonds are observed and Coulomb energies are estimated as shown by

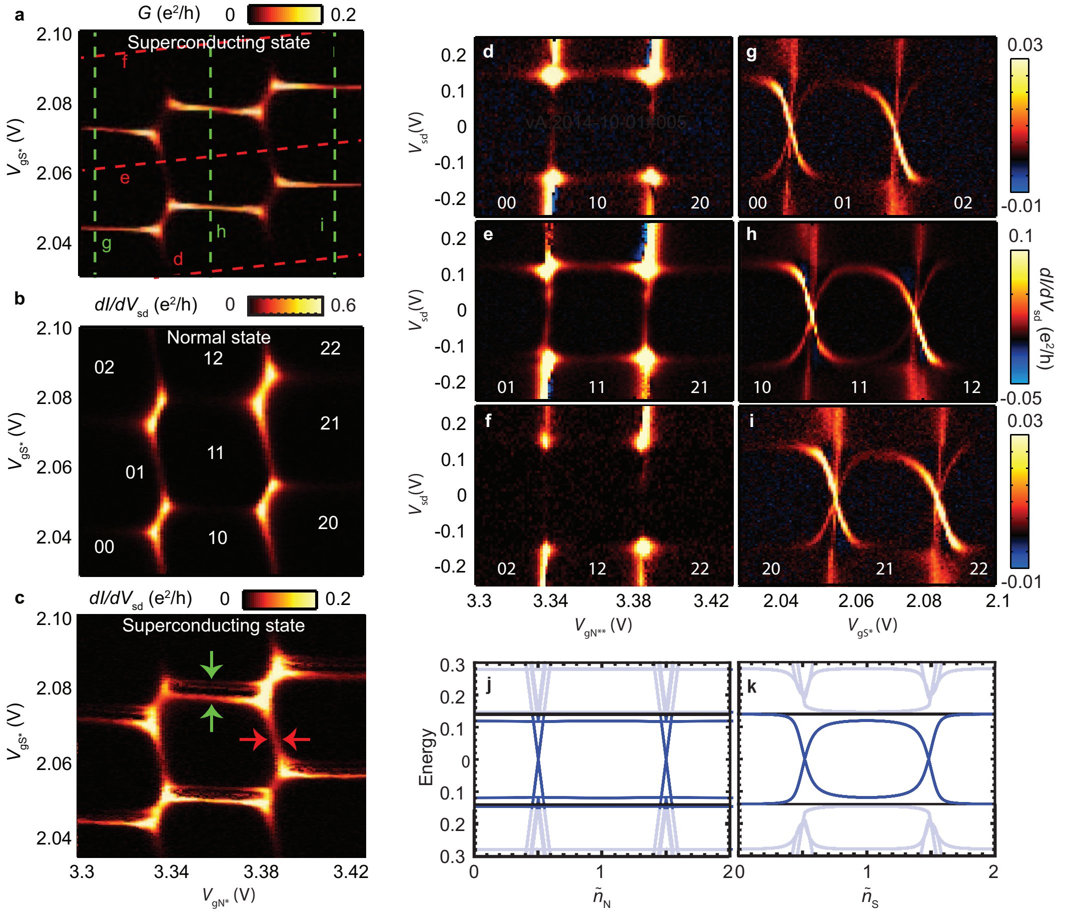

Supplementary Figure 4. Device B: Sub-gap spectroscopy. **a-b**, Stability diagram showing linear conductance versus (combined) plunger gates in the superconducting and normal states. **c**, Stability diagram of the same gate region for a finite bias of $V_{\mathrm{sd}} = 50\,\mu\mathrm{V}$. At finite bias, lines related to sub-gap excitation in $\mathrm{QD_S}$ can be identified by a splitting, which is not present in $\mathrm{QD_N}$ (see green and red arrows, respectively). **d-f**, Gap spectroscopy (bias versus gate) for filling of $\mathrm{QD_N}$ with fixed occupation of $\mathrm{QD_S}$ showing no sub-gap resonances. **g-i**, Gap spectroscopy of $\mathrm{QD_S}$ with fixed occupation in $\mathrm{QD_N}$ showing clear sub-gap resonances. **j-k**, ZBW modeling of the sub-gap excitations energy through the (11) region versus effective voltages $\tilde{n}_i$ on the two dots showing qualitatively good correspondence with experiment (e and h). Parameters are given in Supplementary Table 1.

the dashed diamonds, yielding $U_{\mathrm{S}} = 2.9\,\mathrm{meV}$ and $U_{\mathrm{N}} = 3.1\,\mathrm{meV}$. Finally, the honeycomb stability diagram in the normal state is used to estimate the interdot Coulomb energy $U_{\mathrm{d}} = 0.8$ meV. The couplings t_{S} and t_{N} are found as fitting parameter to the sub-gap

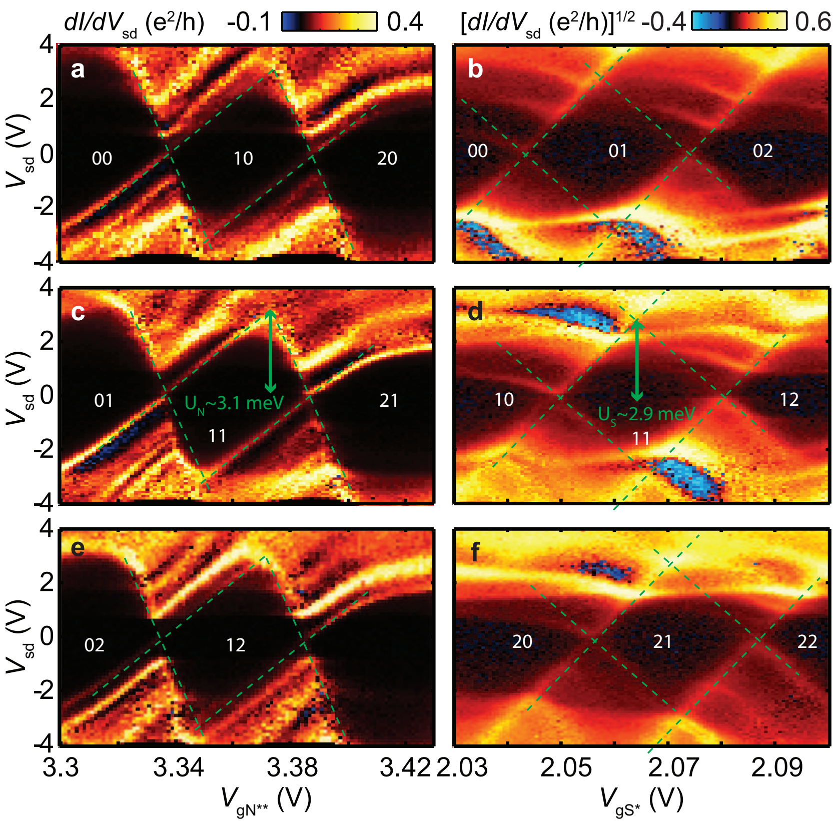

Supplementary Figure 5. Device B: High bias spectroscopy. Bias spectroscopy in the normal state ($B = 170\,\mathrm{mT}$) showing Coulomb blockade diamonds related to Supplementary Fig. 4. **a, c, e**, Bias spectroscopy along the red lines shown in Supplementary Fig. 4**a**, i.e., while changing the occupation in QD_N. Clear Columb diamonds are observed independent of the filling in QD_S yielding a Coulomb energy of $U_N \sim 3.1\,\mathrm{meV}$. **b, d, f**, Coulomb blockade diamonds observed in QD_S versus filling yielding Coulomb energies of $U_S \sim 2.9\,\mathrm{meV}$. The colorscale is the square root of the differential conductance (with sign) to enhance the features at low conductance. The single and double star superscripts on the gate voltages indicate that these correspond to appropriate mixtures of the original gate voltages to separately control the occupancy in QD_N and QD_S.

excitations. The low value of t_N is consistent with the almost straight boundaries in the normal-state honeycomb diagram, i.e., no signature of strong bonding formation between e.g. the (01) and (10) states in Supplementary Fig. 4b.

SUPPLEMENTARY NOTE 3: ZERO-BANDWIDTH APPROXIMATION

In Ref. 1 it was demonstrated that the weak-coupling YSR sub-gap spectrum can be obtained within the zero-bandwidth (ZBW) approximation, in which the BCS lead is replaced

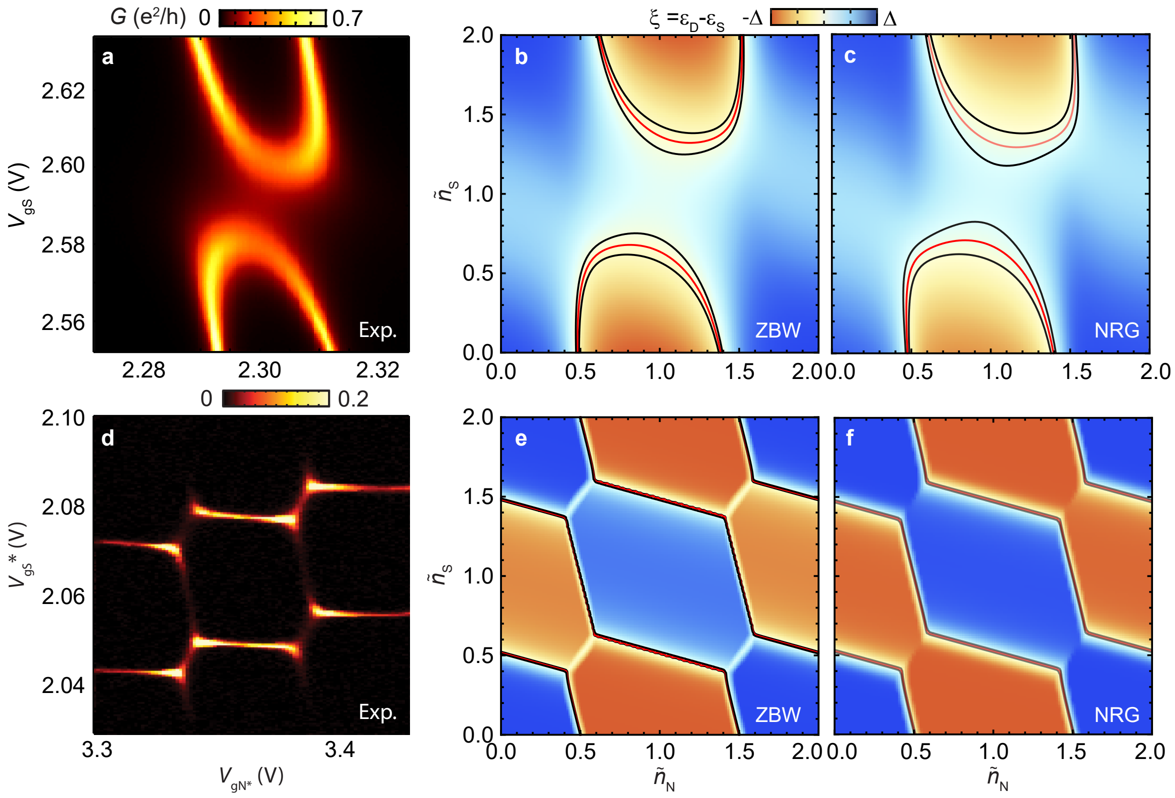

Supplementary Figure 6. Experiment versus model calculations. Comparison between stability diagrams obtained by experiment, ZBW model and NRG calculations. Panels **a, d** show zero-bias conductance versus plunger gate voltages in the partly screened and the honeycomb regime, respectively (measured in two different devices, A and B). Panels **b, e** show the excitation energy, $\xi = \varepsilon_{\mathrm{D}} - \varepsilon_{\mathrm{S}}$, versus dimensionless gate voltage, as modelled within the ZBW approximation in the corresponding regimes. The color scale indicates whether the ground state is a singlet (blue) or a doublet (brown). Red and black contours correspond to zero (degenerate states) and 0.01 meV excitation energy, respectively. Parameters (in meV) are (cf. Supplementary Table 1) $U_{\mathrm{N}} = 2.5$, $U_{\mathrm{S}} = 0.8$, $U_{\mathrm{d}} = 0.1$, $t_{\mathrm{d}} = 0.27$, $t_{\mathrm{S}} = 0.22$, $\Delta = 0.14$ for b) and $U_{\mathrm{N}} = 3.1$, $U_{\mathrm{S}} = 2.9$, $U_{\mathrm{d}} = 0.8$, $t_{\mathrm{d}} = 0.01$, $t_{\mathrm{S}} = 0.1$, $\Delta = 0.14$ for e). Panels **c, f** show NRG calculations using the same parameters except for hybridisation strengths that were fixed at $\Gamma_{\mathrm{S}} = 0.3$ (**c**) and $\Gamma_{\mathrm{S}} = 0.062$ (**f**) that provide the best corresponce to parameters $t_{\mathrm{S}} = 0.22$ and $t_{\mathrm{S}} = 0.1$ in ZBW.

by a single quasiparticle, corresponding to a perturbative solution based on Yosida's variational wavefunction [5, 6]. Here we extend this ZBW approach to deal with the two-orbital Anderson model (1). As we shall see below, the resulting sub-gap spectrum compares very well with the exact results obtained from numerical renormalization group (NRG) calculations, even in the strong coupling regime with a fully YSR screened ground state, and with a number of experimental details observed throughout the 9 different charge states of the stability diagram.

The ZBW approximation corresponds to replacing the Hamiltonian for the S-lead by

$$H_{\mathrm{S}} = \Delta c_{\uparrow}^{\dagger} c_{\downarrow}^{\dagger} + \Delta c_{\downarrow} c_{\uparrow}, \tag{5}$$

where $c_{\sigma}^{\dagger}$ may be thought of as the creation of an electron in the local frontier orbital of the superconducting lead (cf. Appendix C in Ref. 7), which is most strongly coupled to the dot. For now, we focus on the sub-gap spectrum, and the normal lead will be neglected altogether until we return to discuss the actual conductance.

The dot part of the Hamiltonian can be rewritten as

$$H_{\mathrm{D}} = \sum_{i=\mathrm{S/N}} \frac{U_i}{2} \left(n_i - \tilde{n}_i\right)^2 + U_{\mathrm{d}}(n_{\mathrm{S}} - \tilde{n}_{\mathrm{S}})(n_{\mathrm{N}} - \tilde{n}_{\mathrm{N}}) \tag{6}$$

with $n_i = n_{i\uparrow} + n_{i\downarrow}$ and $\tilde{n}_i = 1/2 - \epsilon_i/U_i$. The parameters $\tilde{n}_i$ are hence rescaled gate voltages defined so that in the limit of weakly coupled dots one has $\langle n_i \rangle \approx \tilde{n}_i$. One can also introduce the related parametrisation $\delta_i = 1 + 2\epsilon_i/U_i$ which quantifies the departure from the particle-hole symmetric point at $\delta_i = 0$. The two sets are connected through $\delta_i/2 = 1 - \tilde{n}_i$. The tunneling Hamiltonian reduces to

$$H_{\mathrm{T}} = t_{\mathrm{S}} \sum_{\sigma} c_{\mathrm{S}\sigma}^{\dagger} d_{\mathrm{S}\sigma} + t_{\mathrm{d}} \sum_{\sigma} d_{\mathrm{S}\sigma}^{\dagger} d_{\mathrm{N}\sigma} + \mathrm{h.c.} \tag{7}$$

This simple ZBW Hamiltonian is readily diagonalized numerically. Focusing on the sub-gap spectrum, we may now compare to the experimental data. In Supplementary Fig. 6 we show our best ZBW fit (b, e) to the experiment (a, d). To further verify that the ZBW model indeed yields an acceptable qualitative picture, we also performed NRG calculations and found excellent agreement, see Supplementary Fig. 6(c, f). In addition, we systematically compared ZBW and NRG in different regimes. In Supplementary Fig. 7 the stability diagrams calculated by both methods for different couplings to the superconducting lead show

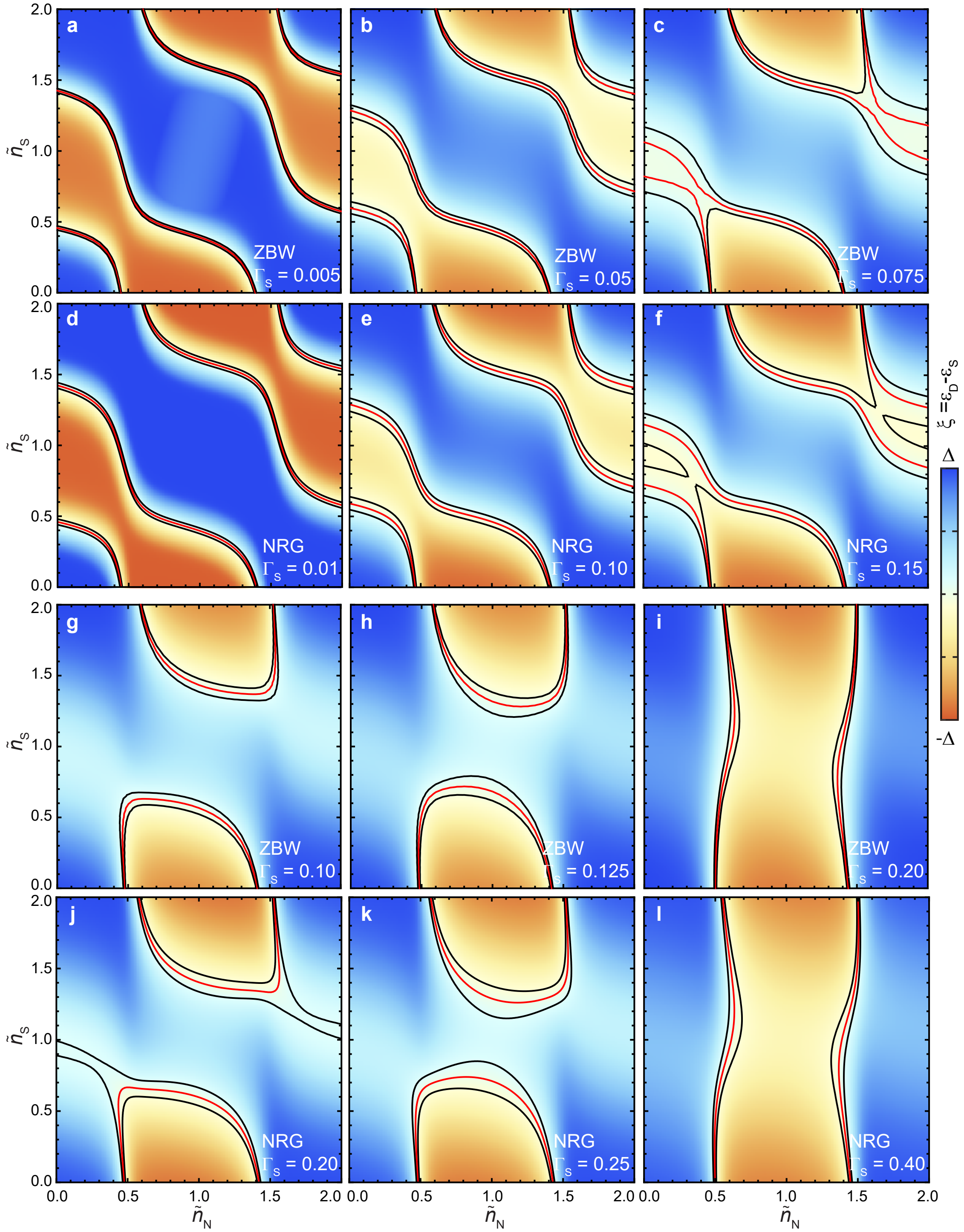

Supplementary Figure 7. Zero-bandwidth versus numerical renormalization group results. Comparison of ZBW (rows 1 and 3) and NRG results (rows 2 and 4) for different couplings to S with $\Gamma_{\rm S} = \nu_{\rm F}\pi t_{\rm S}^2$ in meV, i.e. $t_{\rm S}(\text{meV}) = 0.005,\ 0.05,\ 0.075,\ 0.1,\ 0.125,\ 0.2$ corresponding to (**a, d**),(**b, e**), (**c, f**), (**g, j**), (**h, k**) and (**i, l**). Color scheme and contours as in Supplementary Fig. 6. Parameters used (in meV): $U_{\rm N} = 2.5$, $U_{\rm S} = 0.8$, $U_{\rm d} = 0.1$, $t_{\rm d} = 0.22$, and $\Delta = 0.14$.

the evolution from the honeycomb regime via the partly screened to the screened regime. The contour lines indicate an excitation energy of zero (red) and 0.01 meV (black), respectively, while the colours indicate the ground state (blue for singlet and brown for doublet). It is a priori unclear how to relate the hopping t_{S} in the ZBW to the hybridisation strength Γ in the NRG, since in ZBW the continuum of states is represented by a single level. In our NRG calculations we used a flat density of states with a Γ that does not depend on the energy. Empirically we found that the best agreement between both approaches as regards the positions of the YSR states, which is our main focus here, is obtained for $\Gamma = c\, t_{\mathrm{S}}^2$ with $c \approx 2\pi$.

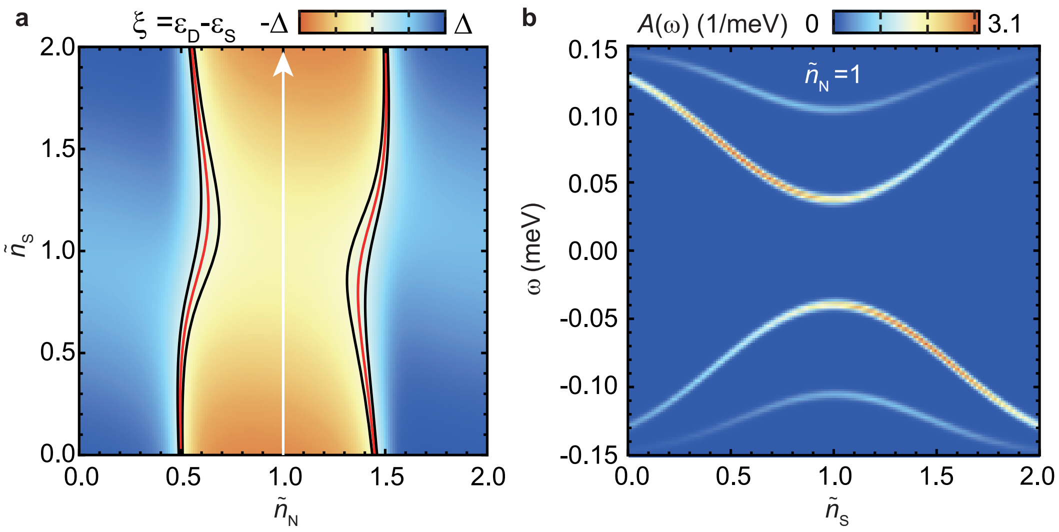

Supplementary Figure 8. Spectral weight of the triplet state. **a**, Stability diagram showing excitation energy versus filling of the two dots obtained by NRG calculations. For these parameters (meV) $U_{\mathrm{N}} = 2.5$, $U_{\mathrm{S}} = 0.8$, $U_{\mathrm{d}} = 0.1$, $t_{\mathrm{d}} = 0.22$, $\Delta = 0.14$, $\Gamma = 0.4$, the YSR doublet is the ground state in the (11) sector and excitations to the singlet (first excited) and triplet (second excited) states are possible. **b**, Spectral density versus filling of $\mathrm{QD_S}$ along the arrow in **a**. The singlet and triplet excitations are seen in the plot with more spectral weight on the singlet than the triplet state. This difference makes it difficult to observe the triplet state in experiments.

Spectral weight of the triplet state

In this section we comment on the fact that the YSR doublet to triplet excitation was not observed in the experiments presented in Fig. 4 in the main text, although the triplet state is theoretically predicted to exist and the transition is allowed by the selection rules. Supplementary Fig. 8a shows the stability diagram in the screened regime, where the ground state changes from singlet (blue) to doublet (brown) to singlet as the electrochemical potential on $\mathrm{QD_N}$ is swept, almost independently of the filling of $\mathrm{QD_S}$. To examine the sub-gap excitation spectrum, we make a cut along the $n_\mathrm{N} = 1$ line shown in Supplementary Fig. 8a by an arrow. The resulting spectral density plot along this line is shown in Supplementary Fig. 8b. The spectral density plot reveals two excitations corresponding to the singlet and triplet states at low and high excitation energy, respectively. It is clearly seen that the triplet excitation has significantly lower spectral weight than the singlet excitation, which may give an indication why the triplet state was not experimentally observed. For finite coupling to N, the sub-gap states broaden and this effect is further pronounced.

SUPPLEMENTARY NOTE 4: SUB-GAP CONDUCTANCE VIA YSR STATES

As described above and in the main text, the ZBW approximation captures the observed YSR sub-gap spectra very well. For not too large couplings, it also agrees remarkably well with NRG calculations, which unlike the ZBW calculations also provide reliable spectral functions. Still, it is the conductance rather than the equilibrium spectral function which is being measured in experiment, a nonequilibrium property which is generally difficult to calculate. In order to address this question, we now perform a Schrieffer-Wolff transformation to obtain an effective Kondo model from which we then calculate the conductance within the polarized-spin approximation, $\mathbf{S} \approx S\hat{z}$, which is known to describe the YSR states quite well [1, 8] (and of course the approximation originally used by Yu, Shiba, and Rusinov [9–11]).

We consider only charge states with $n_i = 0, 1$ and 2 on each dot, and focus here only on $(n_\mathrm{S}, n_\mathrm{N}) = (1, 0)$ and $(n_\mathrm{S}, n_\mathrm{N}) = (1, 2)$. For these charge states $\mathrm{QD_N}$ is kept magnetically inactive and the experimental results look very similar to those of a strongly coupled single dot. In the following treatment we start by setting $U_\mathrm{d} \approx 0$. This results in charge sectors $(1, 0)$ and $(1, 2)$ yielding identical lowest order models, so we will not differentiate between the two. Including fluctuations to the neighboring charge states up to leading orders in t_S^2 and $t_\mathrm{S} t_\mathrm{d} t_\mathrm{N}$, we obtain the following effective exchange-cotunneling Hamiltonian

$$H = H_\mathrm{S} + H_\mathrm{N} + H_\mathrm{K} \tag{8}$$

$$H_\mathrm{S} = \sum_k \left[\sum_\sigma \xi_{\mathrm{S}k} c^\dagger_{\mathrm{S}k\sigma} c_{\mathrm{S}k\sigma} + \Delta (c^\dagger_{\mathrm{S}k\uparrow} c^\dagger_{\mathrm{S}-k\downarrow} + c_{\mathrm{S}-k\downarrow} c_{\mathrm{S}k\uparrow}) \right] \tag{9}$$

$$H_\mathrm{N} = \sum_{k\sigma} \xi_{\mathrm{N}k} c^\dagger_{\mathrm{N}k\sigma} c_{\mathrm{N}k\sigma} \tag{10}$$

$$H_\mathrm{K} = \sum_{kk'\alpha\alpha'} \left[J_{\alpha\alpha'} \sum_{\sigma\sigma'} c^\dagger_{\alpha k\sigma} \mathbf{S} \cdot \sigma_{\sigma\sigma'} c_{\alpha' k'\sigma'} + W_{\alpha\alpha'} \sum_\sigma c^\dagger_{\alpha k\sigma} c_{\alpha' k'\sigma} \right] \tag{11}$$

with $\mathbf{S}$ denoting the spin 1/2 on the S-dot and

$$J_\mathrm{SS} = \frac{t_\mathrm{S}^2}{U_\mathrm{S}} \frac{4}{1-\delta_\mathrm{S}^2}, \quad W_\mathrm{SS} = \frac{t_\mathrm{S}^2}{U_\mathrm{S}} \frac{2\delta_\mathrm{S}}{1-\delta_\mathrm{S}^2}, \quad J_\mathrm{NS} = -\frac{t_\mathrm{S} t_\mathrm{d} t_\mathrm{N}}{U_\mathrm{S} U_\mathrm{N}} \frac{8}{1-\delta_\mathrm{S}^2}, \quad W_\mathrm{NS} = -\frac{t_\mathrm{S} t_\mathrm{d} t_\mathrm{N}}{U_\mathrm{S} U_\mathrm{N}} \frac{4\delta_\mathrm{S}}{1-\delta_\mathrm{S}^2} \tag{12}$$

where $\delta_\mathrm{S} = 1 + 2\epsilon_\mathrm{S}/U_\mathrm{S}$ is the dimensionless deviation from the particle-hole symmetric point. Note that we exclude corrections of order Δ/U_α, U_d/U_α and $t_\mathrm{N}^2/U_\mathrm{N}$. Implementing the polarized-spin approximation by substituting $\mathbf{S} \to 3S\hat{z}$ ensures the correct correspondence

with perturbation theory using the full quantum spin [1], so with $S = 1/2$, we end up with the following tunneling Hamiltonian

$$H_{\mathrm{K}} = \sum_{kk'\alpha\alpha'} \left[\frac{3J_{\alpha\alpha'}}{2} \sum_{\sigma} c^{\dagger}_{\alpha k\sigma} \sigma_z c_{\alpha' k' \sigma} + W_{\alpha\alpha'} \sum_{\sigma} c^{\dagger}_{\alpha k\sigma} c_{\alpha' k' \sigma} \right]. \tag{13}$$

Neglecting J_{NS} and W_{NS}, this Hamiltonian leads to sub-gap YSR bound states with energies $\pm\epsilon_0$ where [1, 8, 11]

$$\epsilon_0 = \pi\nu_{\mathrm{FS}} \frac{1 - \alpha^2 + \beta^2}{\sqrt{(1 - \alpha^2 + \beta^2)^2 + 4\alpha^2}} \tag{14}$$

with $\alpha = 3\pi\nu_{\mathrm{FS}} J_{\mathrm{SS}}/2$ and $\beta = \pi\nu_{\mathrm{FS}} W_{\mathrm{SS}}$, where ν_{FS} denotes the normal-state density of states in the superconductor. Within the polarized-spin approximation, the S-electron self-energy is exact and the Dyson equation is readily solved to find the exact Green's function which has poles at $\omega = \pm\epsilon_0$. Following Refs. 8 and 12, we now restrict the Green's function to $|\omega| < \Delta$ and expand around $\omega \approx \pm\epsilon_0$ to arrive at the retarded 4×4 Nambu Green's function

$$G_0^R(\omega) = \begin{pmatrix} G^R_{0,\uparrow}(\omega) & 0 \\ 0 & G^R_{0,\downarrow}(\omega) \end{pmatrix}, \tag{15}$$

expressed in terms of 2×2 blocks

$$G^R_{0,\uparrow}(\omega) = \frac{1}{\omega + \epsilon_0 + i0_+} \begin{pmatrix} v^2 & uv \\ uv & u^2 \end{pmatrix}, \quad G^R_{0,\downarrow}(\omega) = \frac{1}{\omega - \epsilon_0 + i0_+} \begin{pmatrix} u^2 & -uv \\ -uv & v^2 \end{pmatrix} \tag{16}$$

written in the Nambu-spinor basis

$$\psi^{\dagger}_{\mathrm{S}k\uparrow} = \begin{pmatrix} c^{\dagger}_{\mathrm{S}k\uparrow}, & c_{\mathrm{S}-k\downarrow} \end{pmatrix}, \quad \psi^{\dagger}_{\mathrm{S}k\downarrow} = \begin{pmatrix} c^{\dagger}_{\mathrm{S}k\downarrow}, & -c_{\mathrm{S}-k\uparrow} \end{pmatrix} \tag{17}$$

and with u and v defined as

$$u^2 = 2\pi\nu_{\mathrm{FS}}\Delta \frac{\alpha(1 + (\alpha + \beta)^2)}{((1 - \alpha^2 + \beta^2)^2 + 4\alpha^2)^{\frac{3}{2}}}, \quad v^2 = 2\pi\nu_{\mathrm{FS}}\Delta \frac{\alpha(1 + (\alpha - \beta)^2)}{((1 - \alpha^2 + \beta^2)^2 + 4\alpha^2)^{\frac{3}{2}}}. \tag{18}$$

Notice that, unlike ϵ_0, u and v are sensitive to the sign of β, i.e. to the sign of δ_{S}, which is opposite for gate-voltages on opposite sides of the particle-hole symmetric point, $\epsilon_{\mathrm{S}} = -U_{\mathrm{S}}/2$. Switching sign of δ_{S} effectively interchanges u^2 and v^2. Turning back on the coupling to the normal lead, this effective resonant-level Green's function is readily dressed by cotunneling amplitudes J_{NS} and W_{NS}, and analytically continued to arrive at

$$G^R_{\uparrow}(\omega) = \frac{1}{\omega + \epsilon_0 + i\Gamma} \begin{pmatrix} v^2 & uv \\ uv & u^2 \end{pmatrix}, \quad G^R_{\downarrow}(\omega) = \frac{1}{\omega - \epsilon_0 + i\Gamma} \begin{pmatrix} u^2 & -uv \\ -uv & v^2 \end{pmatrix}, \tag{19}$$

where the total broadening, $\Gamma = \Gamma_\mathrm{r} + \Gamma_\mathrm{e} + \Gamma_\mathrm{h}$, is given in terms of

$$\Gamma_\mathrm{e} = \pi\nu_\mathrm{FN}u^2\left(\frac{3J_\mathrm{NS}}{2} - W_\mathrm{NS}\right)^2, \quad \Gamma_\mathrm{h} = \pi\nu_\mathrm{FN}v^2\left(\frac{3J_\mathrm{NS}}{2} + W_\mathrm{NS}\right)^2. \tag{20}$$

Real parts of the tunneling induced self-energy are neglected here, assuming that ϵ_0 is much smaller than the normal lead bandwidth [13]. The additional term, Γ_r, appearing in the broadening is put in by hand to capture quasiparticle relaxation in the superconductor, as discussed in Refs. 8 and 12. Quasiparticle relaxation could be due to electromagnetic noise or interactions among quasiparticles, say, but here we shall simply model Γ_r as the tunneling rate to an additional fermionic bath with an unspecified bias-independent chemical potential μ_r. Since the problem has been reduced to an effective resonant-level problem, the non-equilibrium Green's functions and the resulting current are readily calculated, and one finds that

$$\begin{aligned} I = \frac{4\mathrm{e}}{h}\int d\omega \Bigg[& \frac{2\Gamma_\mathrm{e}\Gamma_\mathrm{h}n_\mathrm{F}(\omega+eV) - 2\Gamma_\mathrm{e}\Gamma_\mathrm{h}n_\mathrm{F}(\omega-eV)}{(\omega+\omega_0)^2 + (\Gamma_\mathrm{e}+\Gamma_\mathrm{h}+\Gamma_\mathrm{r})^2} \\ & + \frac{\Gamma_\mathrm{r}(\Gamma_\mathrm{h}-\Gamma_\mathrm{e})n_\mathrm{F}(\omega-\mu_\mathrm{r}) + \Gamma_\mathrm{e}\Gamma_\mathrm{r}n_\mathrm{F}(\omega-eV) - \Gamma_\mathrm{h}\Gamma_\mathrm{r}n_\mathrm{F}(\omega+eV)}{(\omega+\omega_0)^2 + (\Gamma_\mathrm{e}+\Gamma_\mathrm{h}+\Gamma_\mathrm{r})^2}\Bigg], \end{aligned} \tag{21}$$

giving the zero-temperature differential conductance

$$\frac{dI}{dV} = \frac{4\mathrm{e}^2}{h}\left[\frac{2\Gamma_\mathrm{e}\Gamma_\mathrm{h} + \Gamma_\mathrm{e}\Gamma_\mathrm{r}}{(V-\epsilon_0)^2 + \Gamma^2} + \frac{2\Gamma_\mathrm{e}\Gamma_\mathrm{h} + \Gamma_\mathrm{h}\Gamma_\mathrm{r}}{(V+\epsilon_0)^2 + \Gamma^2}\right], \tag{22}$$

which no longer contains the unspecified μ_r. When Γ_r vanishes this expression becomes symmetric in bias-voltage, V, since transport is then carried exclusively by Andreev reflections proportional to $2\Gamma_\mathrm{e}\Gamma_\mathrm{h}$ in which incoming electrons are reflected as holes, thereby using both the electron and hole components of the YSR state and transporting two charges (cf. Supplementary Fig. 9b). Notice that aligning the normal-lead Fermi level instead with the negative energy YSR state (labeled v^2 in the figure), it is a Cooper pair at zero energy which splits into the two YSR states and leaves into the empty states of the normal lead. For finite Γ_r, on the other hand, transport may also take place via the bias-asymmetrical relaxation process proportional to $\Gamma_\mathrm{e/h}\Gamma_\mathrm{r}$ in which an electron tunnels from the normal lead to a YSR state, which then relaxes into the quasi-particle continuum (cf. Supplementary Fig. 9a).

Bias asymmetry and quasiparticle relaxation

In this section we fit experimental differential conductance with our classical spin model by using the parameters obtained with ZBW. To estimate the parameters we first analyze

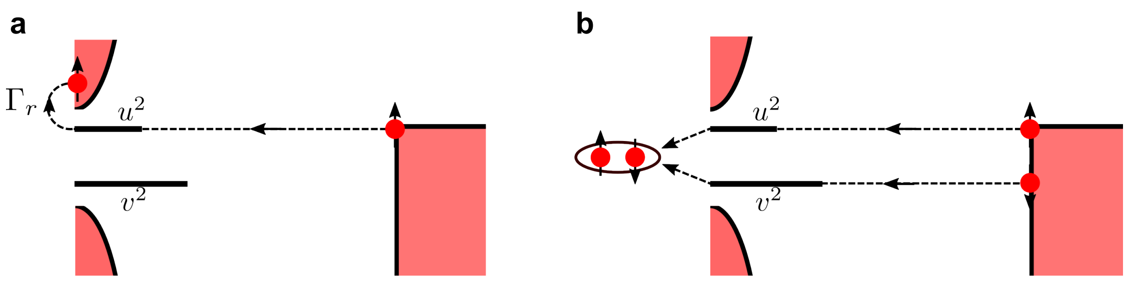

Supplementary Figure 9. Relaxation mechanism. Sketch of the two conducting processes in Supplementary Eq. 22. In **a** we see the relaxation process leading to bias asymmetrical conductance as one can probe the two YSR peaks independently and in **b** the Andreev process which is always bias symmetric as both peaks are always probed together.

the particle-hole symmetric point at which $\Gamma_{\rm e} = \Gamma_{\rm h}$. From Supplementary Eq. 21 we obtain the following finite-temperature conductance for the positive-bias peak,

$$\frac{dI}{dV} = \frac{4{\rm e}^2}{h}\Gamma_{\rm e}\int d\omega \frac{\Gamma}{(\omega-\epsilon_0)^2+\Gamma^2}\frac{dn_{\rm F}(\omega-eV)}{dV} \tag{23}$$

This equation is a convolution of a Lorentzian with a thermal distribution. Experimentally a temperature of $T_{\rm el} \sim 80$ mK $\sim 7\ \mu$V is obtained (we here use a conservative estimate for the electron temperature based on measurements of another sample in the Coulomb blockade regime), while the full width of the YSR state is measured to be approximately 40 μV. For the relevant range of parameters, this integral is very well approximated by adding the temperature to the width of the zero-temperature Lorentzian, that is

$$\frac{dI}{dV} \approx \frac{4{\rm e}^2}{h}\Gamma_{\rm e}\frac{\Gamma+T}{(V-\epsilon_0)^2+(\Gamma+T)^2}, \tag{24}$$

with conductance peak height

$$\left(\frac{dI}{dV}\right)_{\rm max} \approx \frac{{\rm e}^2}{h}\frac{4\Gamma_{\rm e}}{\Gamma+T}. \tag{25}$$

This peak height is measured to be approximately 0.11 e^2/h, which together with the width $2(\Gamma+T) \sim 40\,\mu$V implies that $\Gamma_{\rm e} \sim$ 0.55 μV. Writing out $\Gamma_{\rm e}$ as

$$\Gamma_{\rm e} = u^2(t_{\rm eff}^{\uparrow})^2 = 4\pi\nu_{\rm F}^{\rm N}u^2\frac{t_{\rm S}^2t_{\rm d}^2t_{\rm N}^2}{U_{\rm S}^2U_{\rm N}^2}J^2 = 8\Delta J\frac{\alpha_{\rm S}^2}{(1+\alpha_{\rm S}^2)^2}\frac{t_{\rm d}^2}{U_{\rm S}U_{\rm N}}\pi\nu_{\rm F}^{\rm N}\frac{t_{\rm N}^2}{U_{\rm N}}, \tag{26}$$

we find the following estimate for the dimensionless normal-lead exchange-cotunnel amplitude

$$\nu_{\rm F}^{\rm N}\frac{t_{\rm N}^2}{U_{\rm N}} \sim 0.005, \tag{27}$$

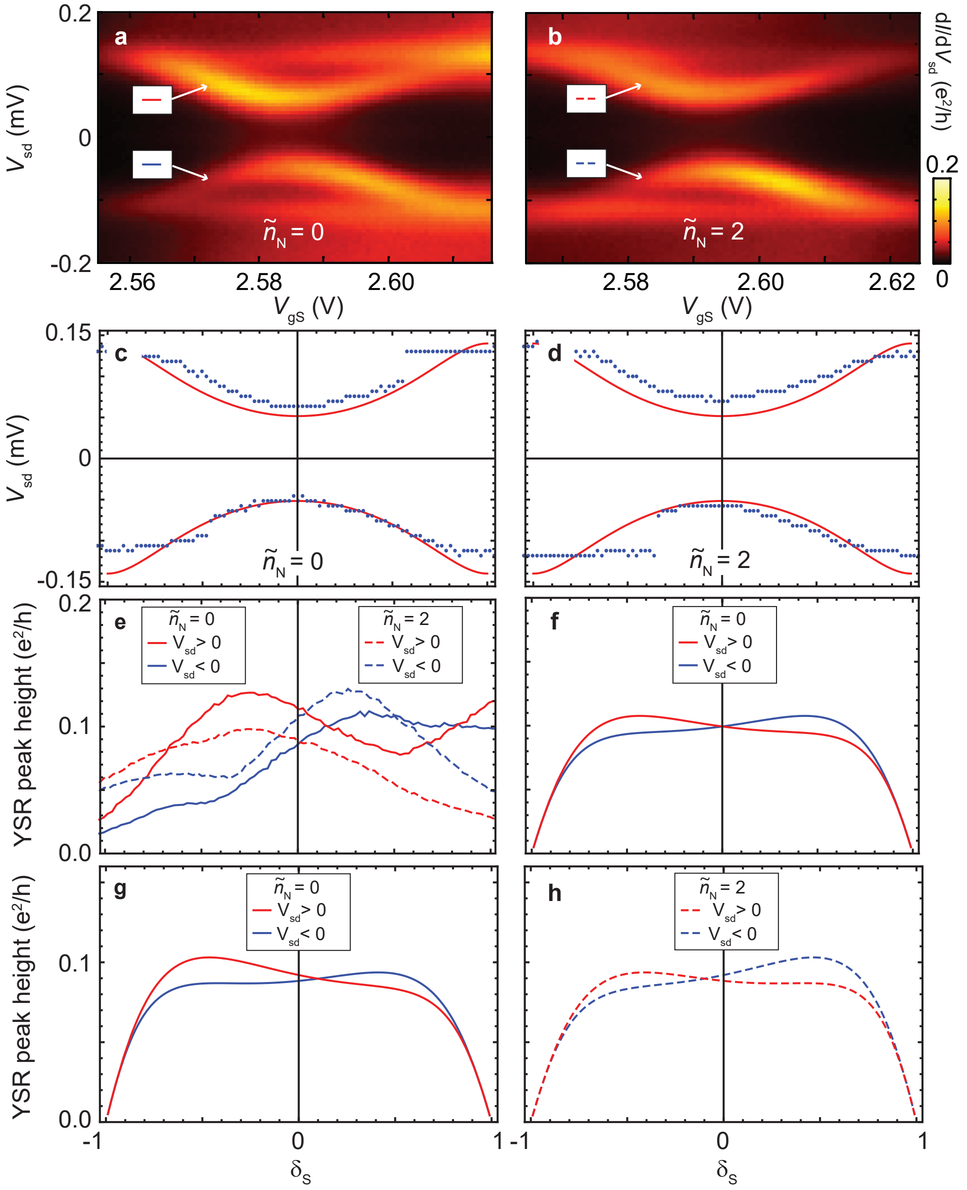

Supplementary Figure 10. Conductance peak shape analysis. **a-b**, Experimental data for $n_{\mathrm{N}} = 0, 2$ and $n_{\mathrm{S}} \in \{0, 2\}$. **c-d**, Experimental conductance peak position in bias voltage plotted as blue dots, and red lines correspond to YSR energies given by Supplementary Eq. 14. **e** Experimental peak conductance as a function of δ_{S}. Red/blue refers to positive/negative bias-voltage and full/dashed to the occupancy of $\mathrm{QD_N}$ being $n_{\mathrm{N}} = 0, 2$. **f-h**, Theoretical plots of peak conductance versus δ_{S} for (1,0) (**f,g**) and (1,2) (**h**), where U_{d} is zero in **f** and $U_{\mathrm{d}} = 0.1$ meV in **g,h**. These are calculated using Supplementary Eq. 23 for the positive-bias peak and interchanging $\Gamma_{\mathrm{e}} \leftrightarrow \Gamma_{\mathrm{h}}$ to obtain the negative-bias peak. The asymmetry observed in experiment is replicated in this model. For all plots **b-h** parameters are chosen to match the parameters (meV) found by zero-bandwidth and NRG ($U_{\mathrm{N}} = 2.5$, $U_{\mathrm{S}} = 0.8$, $t_{\mathrm{d}} = 0.22$, $t_{\mathrm{S}} = 0.25$, $t_{\mathrm{N}} = 0.1$, $\Delta = 0.14$, $\Gamma_{\mathrm{r}} = 0.012$, and $T_{\mathrm{el}} = 80$ mK).

which indicates that we are well away from the Kondo regime, since the Kondo temperature [7] are exponentially suppressed by a factor of $\sim \exp(-1/0.005) \sim 10^{-87}$. Since $\Gamma_{\rm r} = \Gamma - 2\Gamma_{\rm e}$, we may finally estimate the quasiparticle relaxation rate to be $\Gamma_{\rm r} \sim 12\ \mu e$V.

Bias asymmetry at the particle-hole symmetric point.

With this model we are able to explain the observed bias asymmetry by inclusion of a relaxational transport channel, but an additional asymmetry appears in the experimental data. For both $(n_{\rm S}, n_{\rm N}) = (1,0)$ and $(1,2)$, the positive-bias peak conductance is different from the negative-bias peak conductance at the particle-hole symmetrical points, $\delta_{\rm S} = 0$ (cf. Supplementary Fig. 10, panel e). This asymmetry is almost exactly opposite for charge states $(1,0)$ and $(1,2)$, and therefore the explanation is likely to depend on the inter-dot Coulomb interaction, $U_{\rm d}$, by which the charge of the N-dot would effectively gate the S-dot.

Carrying out the Schrieffer-Wolff transformation to arrive at the effective Kondo model (13), we have indeed excluded all corrections of order $U_{\rm d}/U_{\rm N/S}$, and redoing the Schrieffer-Wolff transformation without this omission we find a number of corrections to the cotunnelling amplitudes (12). The second order amplitudes, $J_{\rm SS}$ and $W_{\rm SS}$, are still given by Supplementary Eq. 12 when the N-dot is empty, but with a doubly occupied N-dot, the energy denominators are shifted, and the amplitudes may be obtained from Supplementary Eq. 12 by simply replacing $\delta_{\rm S}$ by $\delta_{\rm S}^{(2)} = \delta_{\rm S} - 4U_{\rm d}/U_{\rm S}$. This corresponds to a shift in the apparent deviation from the particle-hole symmetric point. The third order inter-lead amplitudes, $J_{\rm NS}$ and $W_{\rm NS}$, undergo similar shifts, and expanding to leading order in $U_{\rm d}/U_{\rm N}$, we obtain

$$J_{\rm NS}^{(0)} = J_{\rm NS} + \frac{8t_{\rm S}t_{\rm d}t_{\rm N}U_{\rm d}}{U_{\rm S}U_{\rm N}^2}\left(\frac{1}{1+\delta_{\rm S}+\frac{U_{\rm N}}{U_{\rm S}}} + \frac{1}{1-\delta_{\rm S}}\right), \tag{28}$$

$$W_{\rm NS}^{(0)} = W_{\rm NS} + \frac{4t_{\rm S}t_{\rm d}t_{\rm N}U_{\rm d}}{U_{\rm S}U_{\rm N}^2}\left(\frac{1}{1+\delta_{\rm S}+\frac{U_{\rm N}}{U_{\rm S}}} - \frac{1}{1-\delta_{\rm S}}\right), \tag{29}$$

$$J_{\rm NS}^{(2)} = J_{\rm NS} + \frac{8t_{\rm S}t_{\rm d}t_{\rm N}U_{\rm d}}{U_{\rm S}U_{\rm N}^2}\left(\frac{1}{1+\delta_{\rm S}} + \frac{1}{1-\delta_{\rm S}+\frac{U_{\rm N}}{U_{\rm S}}}\right), \tag{30}$$

$$W_{\rm NS}^{(2)} = W_{\rm NS} + \frac{4t_{\rm S}t_{\rm d}t_{\rm N}U_{\rm d}}{U_{\rm S}U_{\rm N}^2}\left(\frac{1}{1+\delta_{\rm S}} - \frac{1}{1-\delta_{\rm S}+\frac{U_{\rm N}}{U_{\rm S}}}\right), \tag{31}$$

where the superscript refers to the N-dot occupancy. These correction terms are indeed down by a factor of $U_{\rm d}/U_{\rm N}$, but whereas the potential scattering terms are $W_{\rm NS} = 0$ for $\delta_{\rm S} = 0$,

their new correction terms take a finite value. Therefore including $U_{\mathrm{d}}/U_{\mathrm{N}}$ to lowest order in our effective coupling between metal and superconductor renders conductance asymmetric at the particle-hole symmetric point, since the positive peak depends on $\Gamma_{\mathrm{e}} \propto (3/2J_{\mathrm{NS}} + W_{\mathrm{NS}})^2$ and the negative on $\Gamma_{\mathrm{h}} \propto (3/2J_{\mathrm{NS}} - W_{\mathrm{NS}})^2$. Finding peak conductance with this more complete Schrieffer-Wolff transformation yields the same asymmetry as seen in experiment, with positive-bias peaks enhanced and negative-bias peaks suppressed for charge state (1,0), and opposite for (1,2)(cf. Supplementary Fig. 10, panels g,h).

SUPPLEMENTARY REFERENCES

[1] G. Kiršanskas, M. Goldstein, K. Flensberg, L. I. Glazman, and J. Paaske, Phys. Rev. B **92**, 235422 (2015).

[2] A. M. Chang and J. C. Chen, Reports on Progress in Physics **72**, 096501 (2009).

[3] J.-d. Pillet, *Tunneling spectroscopy of the Andreev bound states in a carbon nanotube*, Ph.D. thesis, l'Université Pierre et Marie Curie (2011).

[4] W. G. van der Wiel, S. De Franceschi, J. M. Elzerman, T. Fujisawa, S. Tarucha, and L. P. Kouwenhoven, Rev. Mod. Phys. **75**, 1 (2002).

[5] T. Soda, T. Matsuura, and Y. Nagaoka, Prog. Theor. Phys. **38**, 551 (1967).

[6] K. Yosida, Phys. Rev. **147**, 223 (1966).

[7] A. C. Hewson, *The Kondo Problem to Heavy Fermions* (Cambridge University Press, 1993) (cf. Appendix C).

[8] M. Ruby, F. Pientka, Y. Peng, F. von Oppen, B. W. Heinrich, and K. J. Franke, Phys. Rev. Lett. **115**, 087001 (2015).

[9] L. Yu, Acta Phys. Sin. **21**, 75 (1965).

[10] H. Shiba, Prog. Theor. Phys. **40**, 435 (1968).

[11] A. I. Rusinov, JETP Lett. **9**, 85 (1969), [Zh. Eksp. Teor. Fiz. **9**, 146 (1968)].

[12] I. Martin and D. A. Mozyrsky, Phys. Rev. B **90**, 100508 (2014).

[13] H. Bruus and K. Flensberg, *Many-Body Quantum Theory in Condensed Matter Physics* (Oxford University Press, 2004).
